# Epidemiology of Hepatitis C Virus Among People Who Inject Drugs: Protocol for a Systematic Review and Meta-Analysis

**DOI:** 10.2196/resprot.7936

**Published:** 2017-10-20

**Authors:** Daniel J Smith, Josh Neurer, Ashly E Jordan, Holly Hagan

**Affiliations:** ^1^ Rory Meyers College of Nursing New York University New York, NY United States; ^2^ Center for Drug Use and HIV Research New York University New York, NY United States

**Keywords:** hepatitis C virus, PWID, systematic review, meta-analysis, epidemiology, HCV incidence, HCV prevalence, HCV reinfection

## Abstract

**Background:**

Hepatitis C virus (HCV) is a persistent epidemic among people who inject drugs (PWID), and PWID remain as the population experiencing the most significant impact of HCV-related morbidity and mortality worldwide.

**Objective:**

The purpose of this systematic review and meta-analysis is to synthesize data on the epidemiology of HCV infection among PWID. Our main objectives are to characterize the global and regional distribution and determinants of HCV infection among PWID.

**Methods:**

A search strategy is conducted that involves both the electronic and manual retrievals of literature. Reports are included in this review if they present data published between 2006 and 2015 on prevalent or incident HCV infection among current or former PWID. Standard meta-analytic techniques are performed to synthesize the pooled data and identify correlates of HCV infection.

**Results:**

The search strategy has been performed, and data collection is in progress. Data analysis will follow, and the final results of this systematic review/meta-analysis are expected by December 2017.

**Conclusions:**

This article describes the protocol for the systematic review and meta-analysis of epidemiology of HCV among PWID. We aim to provide synthesized data on HCV incidence and prevalence as well as to identify factors associated with HCV transmission. Our research contributes empirical evidence that informs scholarly, medical, and policy discussions concerning HCV.

**Trial Registration:**

PROSPERO CRD42016035687; https://www.crd.york.ac.uk/prospero/display_record.asp? ID=CRD42016035687 (Archived by WebCite at http://www.webcitation.org/6ttYLn65N)

## Introduction

Hepatitis C virus (HCV) is a persistent epidemic among people who inject drugs (PWID), and PWID remain as the population experiencing the most significant impact of HCV-related morbidity and mortality worldwide [1]. In countries where infection control procedures (which include the systematic screening of blood and blood products) have been implemented, injection drug use is the primary means of HCV transmission [1-2]. Conversely, in countries where procedures to prevent the spread of bloodborne pathogens in health care settings have been less systematically adopted, the spread of HCV has been largely due to nosocomial and iatrogenic causes [3]. Both means of HCV acquisition contribute to an increasing global burden of HCV, and the World Health Organization now estimates that 3% of the world’s population is infected with HCV [4].

Systematic reviews and meta-analyses (SR/MAs) are increasingly being used to inform public health policy and guide allocation of resources to improve population health outcomes. Previous SR/MAs of HCV epidemiology in PWID generated country-specific estimates of prevalent HCV infection (ranging between 10% and 91%) and summarized HCV incidence rates among PWID in Europe (3 to 66/100 person-years) [5-6].

In general, the public health response has not yet matched the pace at which HCV is spreading with respect to primary prevention; HCV prevalence remains high despite the availability of more tolerable and efficacious treatment [7-8]. Additional research and resources are necessary to improve treatment and prevention strategies that address the HCV epidemic among PWID globally. Indeed, individual-level risk behavior during drug injection also has implications for controlling coinfection, especially with human immunodeficiency virus (HIV) and/or hepatitis B virus (HBV).

This article describes the protocol for an SR/MA of the epidemiology of HCV infection among PWID. Our main objectives in the SR/MA are to synthesize global and regional HCV incidence and prevalence and synthesize determinants of HCV infection among PWID.

The SR/MA examines a range of topics on the epidemiology of HCV among PWID. In particular, we assess the following: time to HCV seroconversion subsequent to the onset of drug injection, updating estimates from a previous SR/MA [9]; trends in incidence and prevalence by geographic region; the effect of harm reduction (ie, substance use treatment and syringe/drug injection equipment access programs) on disease burden; and associations between HCV infection and PWID characteristics. Findings in these areas will be interpreted in relation to public health policy.

## Methods

This protocol is consistent with the Preferred Reporting Items for Systematic Reviews and Meta-Analyses Protocol 2015 statement (see Multimedia Appendix 1) [10-11].

### Selection Criteria

Reports are included in this review if they satisfy the following criteria:

- Evaluated participants who reported current or previous injection drug use (ie, PWID)
- Presented original data on HCV prevalence or incidence based on laboratory-confirmed HCV infection in a sample composed of at least 90% PWID
- Published between January 1, 2006, and December 1, 2015

We will exclude reports that are SR/MAs, present self-reported HCV data, or use simulated data.

### Participants

The study population is PWID. We define active PWID as individuals who have injected within the past year, while former PWID are those whose most recent injection drug use occurred over 1 year prior to study admission. Reports that do not provide a detailed description of what constitutes a PWID will be analyzed as a separate group (thus producing 3 categories of PWID: active, former, and status nonspecified). If reports explicitly state that PWID are current or active (or use similar language to convey that PWID have recently injected drugs or are currently injecting drugs) but do not specify a time frame, then we will consider these PWID as active PWID.

### Outcome Measures

The primary outcome is the frequency of HCV infection. We use 4 measures to capture disease distribution:

1. Prevalence of HCV infection
2. Incidence of HCV infection
3. Incidence of HCV reinfection following spontaneous clearance
4. Incidence of HCV reinfection following sustained virologic response to HCV treatment

Prevalence and incidence are measured as proportions and rates, respectively, where possible.

The preferred criteria for measuring prevalent HCV infection are both positive HCV antibody and HCV RNA test results. The alternative criterion is a single positive antibody marker.

Detection of HCV antibody or RNA in a previously seronegative individual are the preferred criteria for determining incident HCV infection. The alternate criterion is RNA positivity in an antibody-negative individual [12].

Measuring incident reinfection subsequent to spontaneous clearance or sustained virologic response is similar. The preferred criterion is a change in the HCV genotype from the cleared infection (either spontaneously or through treatment) to the recently acquired infection over consecutive tests. In the absence of genotype testing, conversion from RNA negative to RNA positive over multiple tests is the alternative criterion.

Secondary outcomes examined are associations between exposures and HCV incidence or prevalence. Exposures include biological and environmental factors such as HIV coinfection, sex, and geographic location.

### Search Strategy

Literature is searched electronically and manually. Electronic searches using a string consisting of terms related to HCV, epidemiology, and PWID are undertaken in 4 databases: Cumulative Index to Nursing and Allied Health Literature, Excerpta Medica database, ProQuest, and PubMed (see Multimedia Appendices 2-5). Results are filtered by date of publication (01/01/2006), record type (peer-reviewed journal), and language (English). Manual searches are performed on reference lists of eligible reports and other relevant papers, conference materials, and research study websites.

### Report Selection

Reports are assessed for inclusion through 3 stages. First, each unique record in the deduplicated set of literature, all of which is retrieved from the search strategy, is screened by title and abstract to determine if it meets the eligibility criteria. Second, for every report that is considered eligible, the full text is subsequently screened. Reports that did not meet the criteria at either of these 2 stages are excluded from further consideration. Third, following data extraction, a final assessment is made on the admissibility of each report.

### Report Appraisal

The quality of reports is determined through the use of an adapted version of the Quality In Prognosis Studies instrument developed by Hayden et al [13-14]. The instrument, which was modified for 2 previous SR/MAs [15-16], evaluates potential sources of bias, such as selection, misclassification, and confounding, in a report; it is also used to evaluate all study designs (eg, cross-sectional, prospective and retrospective cohort, randomized controlled trial).

### Quality Assurance

Two research assistants, both with graduate training in epidemiologic research, systematic review methodology, and biostatistics, perform the literature search and identify eligible reports. Screening and coding pilots are conducted to evaluate intercoder reliability and refine the protocols governing report eligibility and data extraction. The research assistants independently review, code, and appraise all literature, which is subsequently evaluated by the principal investigator and the project director. The principal investigator and project director resolve any issues that emerge throughout the study selection and data extraction processes. The data for all included reports are aggregated into an electronic database that is managed by the research assistants, project director, and principal investigator. The research team is guided by the study protocol that they developed at the start of the project. This SR/MA is registered at PROSPERO [CRD42016035687].

### Data Analysis

The meta-analysis synthesizes report-level data that are collected on the following domains: study cohort, period, and geographic location; study design and methods; HCV incidence, prevalence, and reinfection; and participant characteristics, particularly factors understood to be associated with HCV infection (eg, age, sex, and duration of drug injection). We assess subgroups and the sensitivity of results—determined by report attributes, participant factors, and report quality—and, at each stage, heterogeneity, using Cochran *Q* and *I*^2^. Summary estimates of the primary outcomes are derived using random-effects meta-analysis techniques. Meta-regression models assess the association between characteristics of the study or the sample (eg, location, time period, study quality, safe injection behavior, age, race/ethnicity) and HCV incidence and prevalence. Measures of relative effect are calculated, where possible. Data analysis is conducted in Stata (StataCorp LLC), and data visualization is performed in R (The R Foundation).

## Results

The search strategy has been performed, and data collection is in progress. Data analysis will follow, and the final results of this SR/MA are expected by December 2017.

## Discussion

This article describes the protocol for the SR/MA of the epidemiology of HCV among PWID. We will present global and regional estimates of incident and prevalent HCV infection, and we expect to produce results that identify correlates of incidence and prevalence. Our research contributes empirical evidence that informs scholarly, medical, and policy discussions concerning HCV. Moreover, because the risk behavior of PWIDs is a common route of infection for the transmission of other bloodborne viruses, this review also has implications for research in HIV and HBV.

## Appendix

Multimedia Appendix 1 Preferred Reporting Items for Systematic Reviews and Meta-Analyses Protocol checklist: recommended items to address in a systematic review protocol.

Multimedia Appendix 2 Search strategy for Cumulative Index to Nursing and Allied Health Literature (via EBSCOhost).

Multimedia Appendix 3 Search strategy for Excerpta Medica database (via Ovid).

Multimedia Appendix 4 Search strategy for ProQuest.

Multimedia Appendix 5 Search strategy for PubMed.

Multimedia Appendix 6 Summary statement of the grant and assessment of the study.

## Abbreviations

HBV: hepatitis B virus
HCV: hepatitis C virus
HIV: human immunodeficiency virus
PWID: people who inject drugs
SR/MA: systematic review/meta-analysis
