# Supplementary material for: Epidemiology of Hepatitis C Virus Among People Who Inject Drugs: Protocol for a Systematic Review and Meta-Analysis
Source: JMIR Res Protoc. 2017 Oct 20;6(10):e201. doi: 10.2196/resprot.7936 (PMC5670319; doi:10.2196/resprot.7936)
Supplement: Multimedia Appendix 2 [file resprot_v6i10e201_app2.pdf]

Search no.	Query
1.	MH "Hepatitis C+"
2.	TX "hepatitis c"
3.	TX hcv
4.	TX "hep c"
5.	TX hep* c
6.	TX hepc
7.	TX hepacivirus
8.	TX "hepatitis non a non b"
9.	1 or 2 or 3 or 4 or 5 or 6 or 7 or 8
10.	MH "Epidemiology+"
11.	TI epidemiology or AB epidemiology
12.	TI transmission or AB transmission
13.	MH "Incidence"
14.	TI incidence or AB incidence
15.	MH "Prevalence"
16.	TI prevalence or AB prevalence
17.	MH "Seroconversion"
18.	TX seroconversion
19.	TX seroincidence
20.	MH "Seroprevalence Studies"
21.	TX seroprevalence
22.	TX "re-infection"
23.	TX reinfection
24.	10 or 11 or 12 or 13 or 14 or 15 or 16 or 17 or 18 or 19 or 20 or 21 or 22 or 23
25.	TX "people who inject drugs"
26.	TX PWID
27.	TX IDU
28.	TX IVDU
29.	TX injectors
30.	TX "injecting drug use"
31.	TX "injecting drug user"
32.	TX "injecting drug users"
33.	TX "injecting drug usage"
34.	TX "injection drug use"
35.	TX "injection drug using"
36.	TX "injection drug user"
37.	TX "injection drug users"
38.	TX "injection drug usage"
39.	TX "intravenous drug use"
40.	TX "intravenous drug using"
41.	TX "intravenous drug user"
42.	MH "intravenous drug users"
43.	TX "intravenous drug users"
44.	TX "intravenous drug usage"
45.	TX "parenteral drug use"
46.	TX "parenteral drug user"
47.	TX "parenteral drug users"
48.	MH "Substance Abuse, Intravenous"
49.	TX "intravenous substance abuse"
50.	TX "injection drug abuse"
51.	TX "intravenous drug abuse"
52.	TX "parenteral drug abuse"
53.	TX "drug injection"
54.	MH "Needle Sharing"
55.	TX "needle sharing"
56.	25 or 26 or 27 or 28 or 29 or 30 or 31 or 32 or 33 or 34 or 35 or 36 or 37 or 38 or 39 or 40 or 41 or 42 or 43 or 44 or 45 or 46 or 47 or 50 or 51 or 52 or 53 or 54 or 55
57.	9 and 24 and 56
58.	57. Limiters - Peer Reviewed; Published Date: 20060101-; Exclude MEDLINE records; Language: English
