# Supplementary material for: Epidemiology of Hepatitis C Virus Among People Who Inject Drugs: Protocol for a Systematic Review and Meta-Analysis
Source: JMIR Res Protoc. 2017 Oct 20;6(10):e201. doi: 10.2196/resprot.7936 (PMC5670319; doi:10.2196/resprot.7936)
Supplement: Multimedia Appendix 3 [file resprot_v6i10e201_app3.pdf]

Search no.	Query
1.	exp Hepatitis C virus/ or exp hepatitis C/
2.	hepatitis c.af.
3.	hcv.af.
4.	hep c.af.
5.	hep$ c.af.
6.	hepc.af.
7.	exp Hepacivirus/
8.	hepacivirus.af.
9.	hepatitis non a non b.af.
10.	1 or 2 or 3 or 4 or 5 or 6 or 7 or 8 or 9
11.	exp epidemiology/
12.	epidemiology.ti,ab,kw.
13.	transmission.ti,ab,kw.
14.	exp incidence/
15.	incidence.ti,ab,kw.
16.	exp prevalence/
17.	prevalence.ti,ab,kw.
18.	seroconversion.af.
19.	seroincidence.af.
20.	seroprevalence.af.
21.	re-infection.af.
22.	reinfection.af.
23.	11 or 12 or 13 or 14 or 15 or 16 or 17 or 18 or 19 or 20 or 21 or 22
24.	people who inject drugs.af.
25.	PWID.af.
26.	IDU.af.
27.	IVDU.af.
28.	injectors.af.
29.	injecti$ drug us$.af.
30.	intravenous drug us$.af.
31.	parenteral drug us$.af.
32.	intravenous substance abus$.af.
33.	injection drug abus$.af.
34.	exp intravenous drug abuse/
35.	intravenous drug abus$.af.
36.	parenteral drug abus$.af.
37.	drug injection.af.
38.	needle sharing.af.
39.	24 or 25 or 26 or 27 or 28 or 29 or 30 or 31 or 32 or 33 or 34 or 35 or 36 or 37 or 38
40.	10 and 23 and 39
41.	limit 40 to (english language and yr="2006 -Current" and journal and exclude medline journals)
