# Supplementary material for: Epidemiology of Hepatitis C Virus Among People Who Inject Drugs: Protocol for a Systematic Review and Meta-Analysis
Source: JMIR Res Protoc. 2017 Oct 20;6(10):e201. doi: 10.2196/resprot.7936 (PMC5670319; doi:10.2196/resprot.7936)
Supplement: Multimedia Appendix 4 [file resprot_v6i10e201_app4.pdf]

Search no.	Query
1.	ALL(HCV)
2.	ALL("hep c")
3.	SU.exact.explode("hepatitis c")
4.	ALL("hepatitis c)"
5.	SU.exact.explode(hepacivirus)
6.	ALL(hepacivirus)
7.	ALL(hepc)
8.	ALL("hepatitis non a non b")
9.	1 or 2 or 3 or 4 or 5 or 6 or 7 or 8
10.	SU.exact.explode(epidemiology)
11.	TI(epidemiology) or AB(epidemiology)
12.	SU.exact.(transmission)
13.	TI(transmission) or AB(transmission)
14.	SU.exact.explode(incidence)
15.	TI(incidence) or AB(incidence)
16.	SU.exact.explode(prevalence)
17.	TI(prevalence) or AB(prevalence)
18.	SU.exact(seroconversion)
19.	ALL(seroconversion)
20.	ALL(seroincidence)
21.	ALL(seroprevalence)
22.	ALL("re-infection")
23.	ALL(reinfection)
24.	10 or 11 or 12 or 13 or 14 or 15 or 16 or 17 or 18 or 19 or 20 or 21 or 22 or 23
25.	ALL("people who inject drugs")
26.	ALL(PWID)
27.	ALL(IDU)
28.	ALL(IVDU)
29.	ALL(injectors)
30.	ALL("injecting drug use")
31.	ALL("injecting drug user")
32.	ALL("injecting drug users")
33.	ALL("injecting drug usage")
34.	ALL("injection drug use")
35.	ALL("injection drug using")
36.	ALL("injection drug user")
37.	ALL("injection drug users")
38.	ALL("injection drug usage")
39.	ALL("intravenous drug use")
40.	ALL("intravenous drug using")
41.	ALL("intravenous drug user")
42.	ALL("intravenous drug users")
43.	ALL("intravenous drug usage")
44.	ALL("parenteral drug use")
45.	ALL("parenteral drug user")
46.	ALL("parenteral drug users")
47.	SU.exact("Substance Abuse Intravenous")
48.	ALL("intravenous substance abuse")
49.	ALL("injection drug abuse")
50.	ALL("intravenous drug abuse")
51.	ALL("parenteral drug abuse")
52.	SU.exact("drug injection")
53.	SU.exact("needle sharing")
54.	ALL("needle sharing")
55.	25 or 26 or 27 or 28 or 29 or 30 or 31 or 32 or 33 or 34 or 35 or 36 or 37 or 38 or 39 or 40 or 41 or 42 or 43 or 44 or 45 or 46 or 47 or 50 or 51 or 52 or 53 or 54
56.	9 and 24 and 55
57.	56 and STYPE("scholarly journals") and PY(2006-2015) and LN(english)
