# Supplementary material for: Epidemiology of Hepatitis C Virus Among People Who Inject Drugs: Protocol for a Systematic Review and Meta-Analysis
Source: JMIR Res Protoc. 2017 Oct 20;6(10):e201. doi: 10.2196/resprot.7936 (PMC5670319; doi:10.2196/resprot.7936)
Supplement: Multimedia Appendix 5 [file resprot_v6i10e201_app5.pdf]

Search no.	Query
1.	hcv[All Fields]
2.	"hep c"[All Fields]
3.	"hepatitis c"[MeSH Terms]
4.	"hepatitis c"[All Fields]
5.	"hepacivirus"[MeSH Terms]
6.	"hepacivirus"[All Fields]
7.	hepc[All Fields]
8.	"hepatitis non a, non b"[All Fields]
9.	"hepatitis non a non b"[All Fields]
10.	1 or 2 or 3 or 4 or 5 or 6 or 7 or 8 or 9
11.	"epidemiology"[MeSH Terms]
12.	epidemiology[ot]
13.	epidemiology[tiab]
14.	transmission[ot]
15.	transmission[tiab]
16.	"incidence"[MeSH Terms]
17.	incidence[ot]
18.	incidence[tiab]
19.	"prevalence"[MeSH Terms]
20.	prevalence[ot]
21.	prevalence[tiab]
22.	seroconversion[All Fields]
23.	seroincidence[All Fields]
24.	seroprevalence[All Fields]
25.	re-infection[All Fields]
26.	reinfection[All Fields]
27.	11 or 12 or 13 or 14 or 15 or 16 or 17 or 18 or 19 or 20 or 21 or 22 or 23 or 24 or 26
28.	"people who inject drugs"[All Fields]
29.	PWID[All Fields]
30.	IDU[All Fields]
31.	IVDU[All Fields]
32.	injectors[All Fields]
33.	"injecting drug use"[All Fields]
34.	"injecting drug user"[All Fields]
35.	"injecting drug users"[All Fields]
36.	"injecting drug usage"[All Fields]
37.	"injection drug use"[All Fields]
38.	"injection drug using"[All Fields]
39.	"injection drug user"[All Fields]
40.	"injection drug users"[All Fields]
41.	"injection drug usage"[All Fields]
42.	"intravenous drug use"[All Fields]
43.	"intravenous drug using"[All Fields]
44.	"intravenous drug user"[All Fields]
45.	"intravenous drug users"[All Fields]
46.	"intravenous drug usage"[All Fields]
47.	"parenteral drug use"[All Fields]
48.	"parenteral drug user"[All Fields]
49.	"parenteral drug users"[All Fields]
50.	"Substance Abuse, Intravenous"[Mesh]
51.	"intravenous substance abuse"[All Fields]
52.	"injection drug abuse"[All Fields]
53.	"intravenous drug abuse"[All Fields]
54.	"parenteral drug abuse"[All Fields]
55.	"drug injection"[All Fields]
56.	"Needle Sharing"[Mesh]
57.	28 or 29 or 30 or 31 or 32 or 33 or 34 or 35 or 36 or 37 or 38 or 39 or 40 or 41 or 42 or 43 or 44 or 45 or 46 or 47 or 50 or 51 or 52 or 53 or 54 or 55
58.	10 and 27 and 57
59.	58. Filters: Journal Article; Publication date from 2006/01/01; English
